# From silos to synergy: assessing tuberculosis basic management units readiness for integrated diabetes mellitus care across different districts of Pakistan

**DOI:** 10.1017/S146342362610111X

**Published:** 2026-04-10

**Authors:** Saima Aleem, Saima Afaq, Bilal Ahmad, Zala, Haroon Latif Khan, Zohaib Khan

**Affiliations:** 1 Institute of Public Health & Social Sciences, Khyber Medical University, Pakistan; 2 University of York Department of Health Sciences, UK; 3 Office of Research Innovation and Commercialization, Khyber Medical University, Pakistan; 4 Government of Khyber Pakhtunkhwa Health Department, Pakistan

**Keywords:** Comorbidity, diabetes mellitus, implementation science, integrated care, organizational readiness, tuberculosis

## Abstract

**Background:**

The dual burden of tuberculosis (TB) and diabetes mellitus (DM) presents a growing challenge for health systems in low- and middle-income countries (LMICs), including Pakistan. Despite global and national policies advocating for integrated care, evidence on health facility readiness to operationalize integration remains scarce. This study assessed the readiness of TB basic management units (BMUs) to deliver integrated TB-DM care and explored implementation barriers using the Consolidated Framework for Implementation Research (CFIR).

**Methods:**

We conducted an explanatory sequential mixed-methods study from September 2024 to February 2025 across 13 TB BMUs in five districts of Pakistan. Quantitative readiness data were collected using a structured tool adapted from the WHO Service Availability and Readiness Assessment (SARA), generating a composite score across four domains. Subsequently, qualitative data were gathered through multi-stakeholder focus group discussions with healthcare providers, facility managers, patients, caregivers, and policymakers. Reflexive thematic analysis was conducted and mapped to CFIR Inner Setting constructs to contextualize quantitative findings.

**Results:**

Only one facility demonstrated high readiness, while 12 showed low readiness. Facilities lacked routine comorbidity screening, trained staff, diagnostic capacity, and essential medicines. Key barriers included inadequate infrastructure, workforce shortages, fragmented information systems, and low prioritisation of integrated care. Financial constraints and limited coordination further hindered implementation.

**Conclusion:**

This study highlights critically low readiness among TB facilities in different districts of Pakistan to deliver integrated TB-DM care, reflecting systemic weaknesses across core domains. Strengthening systems, building capacity, and improving integration strategies are essential to bridge gaps between policy and practice.

## Introduction

The global burden of tuberculosis (TB) and diabetes mellitus (DM) represents a growing syndemic in low- and middle-income countries (LMICs), where the dual disease burden has intensified the challenges of health system preparedness and delivery of patient-centred care(Bibi et al., 2025). Three countries, India (26%), Pakistan (6.3%), and Bangladesh (3.5%), account for 35.8% of the global total for TB(WHO, 2024), and DM prevalence in this South Asian region has increased to 22.30% in 2020–2024 (Ranasinghe et al., 2024). With over 95% of TB mortality occurring in LMICs and the increasing prevalence of type 2 DM in these settings, the intersection of these two diseases has emerged as a critical public health issue that undermines traditional models of care (McMurry et al., 2019). Considering the global prevalence of TB-DM comorbidity ranging from 13.73% to 15.3% (Boadu et al., 2024), the bidirectional relationship between TB and DM not only complicates diagnostic and therapeutic processes but also imposes significant additional costs on health systems that are already constrained by limited resources and fragmented service delivery networks (Chamba et al., 2024).

The ‘Collaborative Framework for Care and Control of Tuberculosis and Diabetes’ introduced by World Health Organization (WHO) and the International Union Against Tuberculosis and Lung Disease (The Union) (WHO, 2011) and different integrated care models (USAID, 2020; Mpagama et al., 2021) offer a promising pathway for integration to mitigate the adverse clinical and socioeconomic outcomes associated with TB-DM comorbidity, yet the readiness of health facilities to implement such models remains heterogeneous and generally suboptimal in resource-limited settings (Foo et al., 2022). In many LMICs, the rapid scale-up of vertical TB programmes has not been matched by commensurate investments in DM care infrastructure, resulting in gaps in screening, timely diagnosis, and integrated management that exacerbate treatment losses and adverse treatment outcomes (Nyirenda et al., 2022). This imbalance not only delays the detection and treatment of DM among TB patients but also contributes to higher rates of treatment failure (approx. 4.8% treatment failure in TB-DM patients than 1.5% in non-diabetic TB patients) (Zheng et al., 2017), relapse, and mortality (Jackson-Morris et al., 2024).

Recent strategic initiatives, including the WHO Collaborative Framework and various national policies, have sought to address these challenges through bidirectional screening and integrated approaches that leverage existing TB infrastructure for the management of chronic non-communicable diseases (NCD) (Milice et al., 2024). Nonetheless, evidence indicates that health facility readiness remains compromised by structural deficiencies, including insufficient human resource capacity, limited availability of diagnostics and essential medications, and inadequate training of healthcare providers in integrated management strategies (Shayo and Shayo, 2019).

Lessons from pilot projects in India and South Africa further illustrate that when integrated screening and treatment models are implemented effectively, they can lead to increased case detection, better glycemic control, and improved overall TB treatment outcomes (Fazaludeen Koya et al., 2022; Foo et al., 2022). Despite these promising strategies, persistent barriers continue to hinder the effective integration of DM care within TB services. Critical challenges include the scarcity of trained personnel adept at managing comorbid conditions, frequent interruptions in the supply chain for essential medications, and fragmented information systems that impede timely data sharing and patient follow-up (Salifu and Hlongwana, 2020; Nyirenda, 2023). These issues are compounded by socioeconomic determinants such as poverty, limited health literacy, and geographical barriers that further restrict access to integrated services for the most disadvantaged patients (Salato and Setswe, 2024).

A critical aspect of implementing integrated TB-DM care is the systematic assessment of health facility readiness. The readiness analysis determines the existing strengths and capacities and helps in the identification of gaps to focus on. Such assessments are integral to developing tailored interventions that can optimize the integration process, enhance screening coverage, and ultimately improve patient outcomes.

In the context of Pakistan, the country faces a high dual burden of TB and DM, yet our health system largely continues to operate through vertical, disease-specific programmes. Despite the availability of global and national guidelines for integrated TB-DM care, the implementation of these guidelines remains inconsistent, fragmented, and poorly evaluated, as no baseline readiness assessment of health facilities was done before the introduction of the intervention focusing TB-DM integration. In Pakistan, TB care is delivered through a network of Basic Management Units (BMUs), which serve as the primary operational platform for TB diagnosis, treatment, and follow-up. In this context, our study, being the first of its kind, aimed to evaluate the current state of TB BMUs readiness for the integrated delivery of TB-DM care in selected districts of Pakistan, utilizing a mixed-method approach to providing a comprehensive assessment of the operational landscape. With this study, our objective was to (1) quantify TB basic management units (BMUs) readiness for integrated TB-DM services across four SARA-derived domains; and (2) explain observed readiness through qualitative inquiry mapped to Consolidated Framework for Implementation Research (CFIR) Inner Setting.

## Method

### Study settings

**Figure 1.**
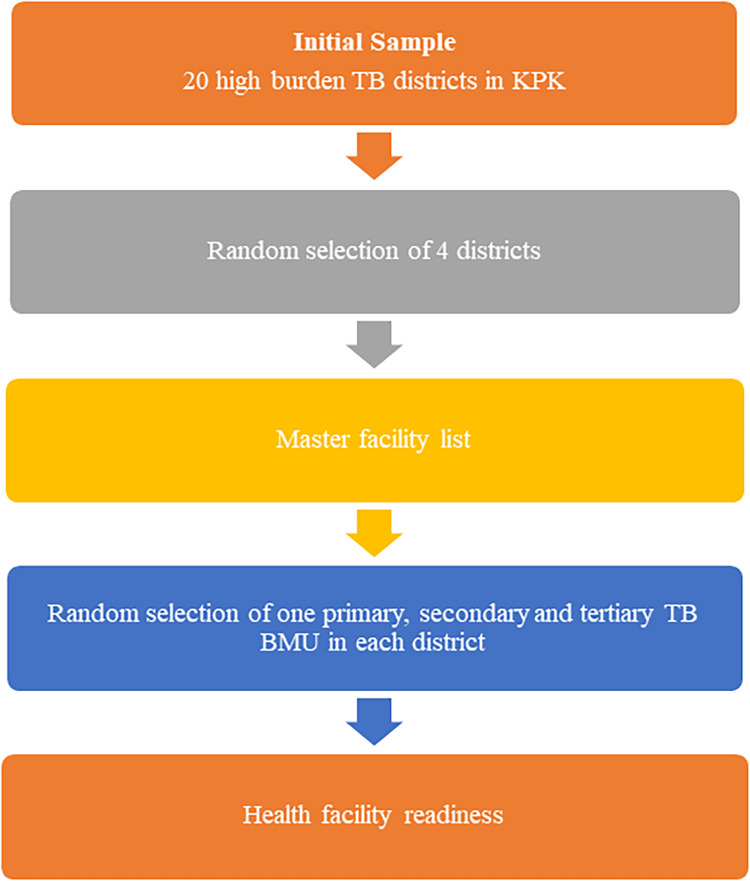
Selection of TB basic management units in KPK for readiness assessment.

We conducted this explanatory sequential mixed-method study between September 2024 and February 2025 at 13 TB BMUs across primary, secondary, and tertiary levels of care, in five districts across two provinces in Pakistan. The integration of DM care in existing TB services in Pakistan is still in its infancy. Interestingly, two distinct provincial profiles emerged while overlaying the provincial incidences on DM prevalence: Khyber Pakhtunkhwa (KPK) (highest TB but still-moderate DM) and Punjab (highest DM but lower, though still considerable, TB) (Hasan and Siddiqui, 2024). In KPK, we collaborated with the Deputy Director of the Provincial TB Program to select facilities. From the 20 high-burden TB districts, we randomly selected four: Peshawar, Mardan, Abbottabad, and Swat (Figure 1). Additionally, we purposively selected the Rawalpindi district from Punjab, where the TB-DM integrated care implementation was planned. Within each selected district, we collaborated with the Provincial TB Program KPK to randomly select public facilities, one facility (TB BMU) each from primary, secondary, and tertiary levels using the district master facility list (Afaq et al., 2024). All the primary facilities were in peri-urban settings, whereas secondary and tertiary level BMUs were located in urban settings. The primary TB BMUs had more rural adjoining area and rural catchment population (further details regarding positionality and functionality of all TB BMUs are added in supplementary file 1). Initially, a total of 15 TB BMUs were selected; however, we excluded two facilities before the initiation of data collection due to an unstable law-and-order situation, making them hard to reach for facility assessment.

### Measures

#### Outcome

**Figure 2.**
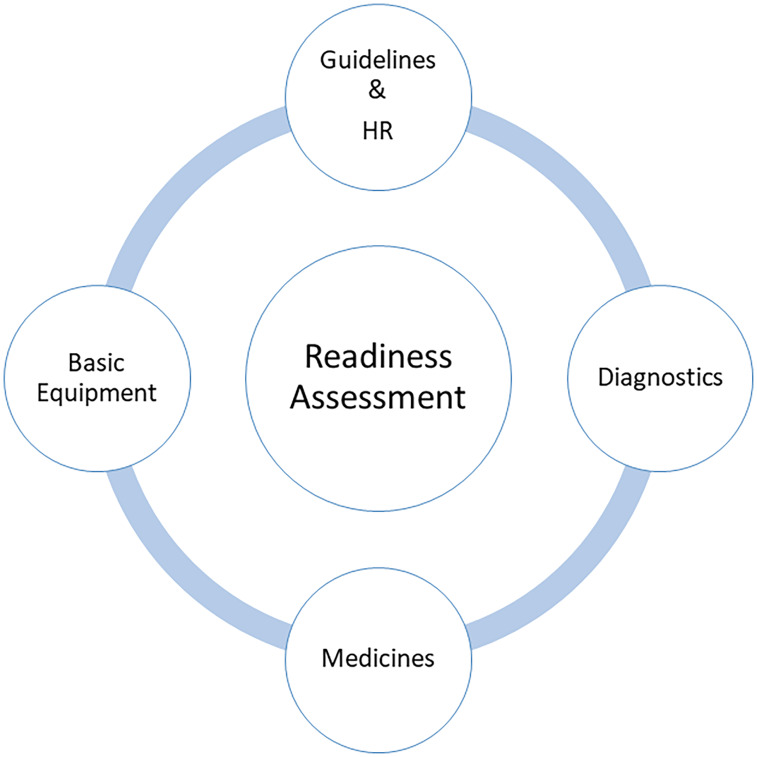
Core domains of TB facility readiness assessment for integrated DM care.

In our study, ‘readiness’ of the TB health facility to manage DM (the service) was the outcome variable. We defined and operationalized readiness as the ‘functional capacity of TB healthcare facilities to deliver DM management’. It was operationalized as a composite readiness score based on the WHO’s Service Availability and Readiness Assessment (WHO-SARA) framework, adapted to focus on NCD integration within TB services. The composite scores were derived from the fourteen tracer indicators divided across four domains (with each domain contributing 0–25 points) as presented in Figure 2.

#### Adaptation of the WHO-SARA tool

**Figure 3.**
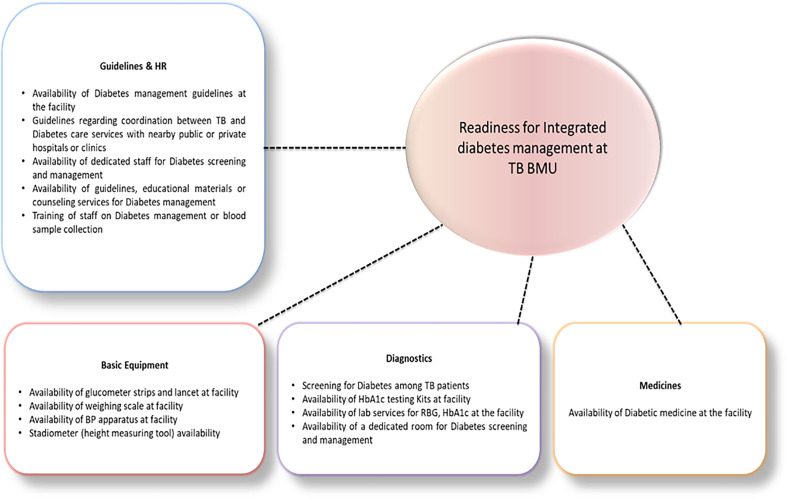
Readiness domains and tracer items used to assess TB-DM integration capacity.

We employed the expert validation process to ensure the contextual relevance and content validity of items in the domains. We involved public health experts, TB programme managers, health care practitioners, and medical educationists familiar with the service readiness in Pakistan. Based on their feedback, minor contextual adaptations were made to the items; however, no structural changes were made to the core domains of the WHO-SARA framework. Expert validation was followed by a pilot test in three health facilities not included in the final readiness assessment. The purpose of this pilot was to assess the clarity and feasibility of administering the tool to capture required information, particularly ensuring that questions were well-understood and appropriately interpreted. No formal reliability testing (e.g., Cronbach’s alpha) was conducted at this stage, as the primary aim was refinement for clarity rather than psychometric validation. The indicators across each domain are presented in Figure 3. We distinguished availability from functionality and applied uniform verification windows. *Dedicated staff* required a documented TB-DM focal person and presence on the visit day; *trained staff* required verifiable TB-DM training within 12 months; *guidelines* required a current, in-use document at the BMU; *glucometer readiness* required a functional device on the day plus compatible strips in stock with no stock-out in the prior 30 days; *HbA1c access* required on-site testing or a formalized referral pathway (lab contracts, named contact, blood sample transport log, timely result return); *medicines* (e.g., metformin/insulin) required physical availability on the day and no stock-out in the prior 30 days (or a documented, reliable supply pathway for insulin). *Referral coordination* required a written logbook with named counterpart(s) and ≥1 documented DM referral in the last quarter.

#### Explanatory variable

Health facilities were categorized as primary, secondary, and tertiary TB BMU due to their functionality and location being categorized by the district where the facilities were located.

#### Qualitative methods

We conducted two multi-stakeholders focus group discussions (FGDs) to elicit explanatory mechanisms underlying the quantitative readiness findings. Participants included representatives from all 13 facilities, including TB Directly Observed Treatment Short-course (DOTS) facilitators and medical officers (MOs), TB patients, their carers, endocrinologists, pulmonologists, TB experts, and managers, community members, researchers, and policy makers. Participants were purposively invited to ensure coverage of districts and care levels. Our focus groups included around 10 participants each. While 6–10 participants are generally recommended for focus groups, we intentionally used the upper end of this range to ensure representation from multiple cadres and districts. The facilitation approach using structured prompts, round-robin contributions, and active probing helped maintain balanced participation. FGDs were conducted in participants’ preferred language and guided by a semi-structured agenda aligned to TB-DM integration domains. With written informed consent, discussions were audio-recorded, supplemented by contemporaneous field notes, and transcribed verbatim. To mitigate dominance, facilitators (SAL, BA, and Z) used round-robin prompts and probed for divergent views, with ground rules that encouraged dissent and confidentiality.

The qualitative team comprised three researchers (SAL, BA, Z) trained in implementation science and health systems. Positionality statements and reflexive memos documented assumptions and their evolution during analysis. Coding and theme development were led by one analyst (SAL) with peer debriefs at multiple points; differences in interpretation were resolved through discussion rather than inter-coder reliability statistics, consistent with RTA. We used data–method triangulation with quantitative results to enhance credibility. Quotations are attributed using anonymized FGD labels to participants to preserve confidentiality while indicating discussion context.

### Data analysis method

The item responses across all domains were categorized as ‘yes’ and ‘no’. We calculated the score of each domain using SPSS V 29 by summing the score of each item and dividing it by the total number of items in that domain. Following the WHO-SARA reference manual, the composite score was calculated based on the aggregates. Following prior studies, we assigned equal weight to each domain (25% each) and calculated overall readiness as the mean of the four domain indices. Facilities scoring ≥50% were classified as high readiness, and those scoring <50% as low readiness (Agwanda et al., 2009; Wang et al., 2017; Bintabara and Mpondo, 2018; Shayo and Shayo, 2021).

We analysed data from the FGDs using reflexive thematic analysis (Braun and Clarke, 2006) to identify key barriers and challenges. Analysis proceeded through the following phases: familiarization by repeated reading, coding, generating initial themes, reviewing themes, defining and naming themes, and producing the final analytic details by selecting illustrative quotations and integrating with quantitative results. Following reflexive thematic analysis, we did a structured mapping to CFIR to locate each theme within relevant inner setting constructs. Themes were generated inductively and then positioned deductively relative to CFIR to structure findings within an established implementation science framework.

### Synthesis of determinant themes within the CFIR inner setting domain

We selected the CFIR to guide the mapping of implementation determinants identified through the consultative meetings, with a particular focus on the Inner Setting domain (Damschroder et al., 2022). CFIR provides a comprehensive and systematic structure to explore, categorize, and interpret factors that influence the implementation of complex health interventions within organizations. The inner setting domain specifically encompasses the organizational characteristics, capacities, and contextual factors that shape how new practices can be adopted, integrated, and sustained within healthcare environments. In the context of our study, the integration of comorbid DM care into existing TB services in Pakistan presents multifaceted implementation challenges. These challenges are deeply rooted in organizational infrastructure, resources, leadership engagement, communication networks, and the prevailing implementation climate.

### Triangulation of data

Given the explanatory sequential mixed-method design, we employed triangulation to strengthen the validity, credibility, and depth of our findings. Quantitative data quantified health facility readiness for integrated TB-DM care, while qualitative data highlighted the underlying organizational, operational, and contextual barriers shaping these readiness outcomes. Triangulating these data strands allowed for a comprehensive synthesis, enhancing both the explanatory power of the quantitative findings and the robustness of conclusions drawn for practice and policy.

For the triangulation, we followed a systematic and domain-based approach and mapped quantitative and qualitative findings to four WHO-SARA-derived readiness domains. Qualitative data were further mapped to the CFIR inner setting construct to provide explanatory depth. Patterns of convergence, divergence, and complementarity were identified and synthesized to produce integrated insights.

## Results

Of the 13 health facilities, four were primary care, five were secondary, and four were tertiary care TB BMUs. These BMUs varied by district: Peshawar had two primary, one secondary, and one tertiary facility, while Mardan and Abbottabad each had one facility at all three levels. Rawalpindi had one primary and one secondary facility, and Swat contributed a single secondary facility (two facilities were dropped due to the law-and-order situation).

## Readiness of TB health facility for integrated DM care & management

Overall, the readiness of TB BMU to provide integrated DM care and management was low (92.3%) with composite scores ranging from 15.0 to 60.4. The relationship map illustrates the interconnectedness of health facilities across the selected districts (Rawalpindi, Swat, Mardan, Abbottabad, and Peshawar) in relation to their levels of service readiness, categorized as low readiness and high readiness.

**Figure 4.**
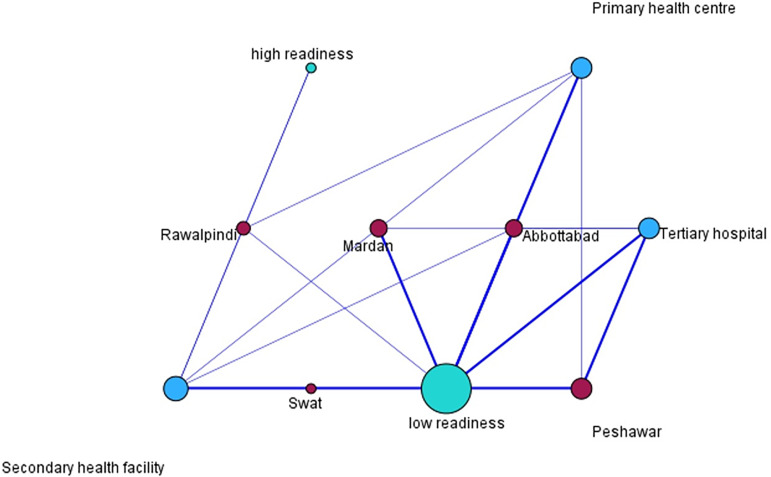
Diabetes mellitus care related readiness level relational map across selected TB BMUs.

A key observation from the relational map (Figure 4) is the predominant clustering around low readiness, which is visually represented by the larger central node labelled ‘low readiness’. This indicates that a substantial proportion of facilities, particularly within Mardan, Abbottabad, Swat, and Peshawar, exhibit strong associations with limited readiness to deliver integrated services. The thickness of the connecting lines further reinforces these associations, highlighting persistent systemic weaknesses that transcend facility type and geographical boundaries.

Conversely, high readiness is depicted as a peripheral and isolated occurrence, primarily associated with a single facility in Rawalpindi. The relatively smaller size of this node and its limited number of connections suggest that high readiness is not a common characteristic within the current network of facilities, and its influence on the overall system remains marginal.

**Figure 5.**
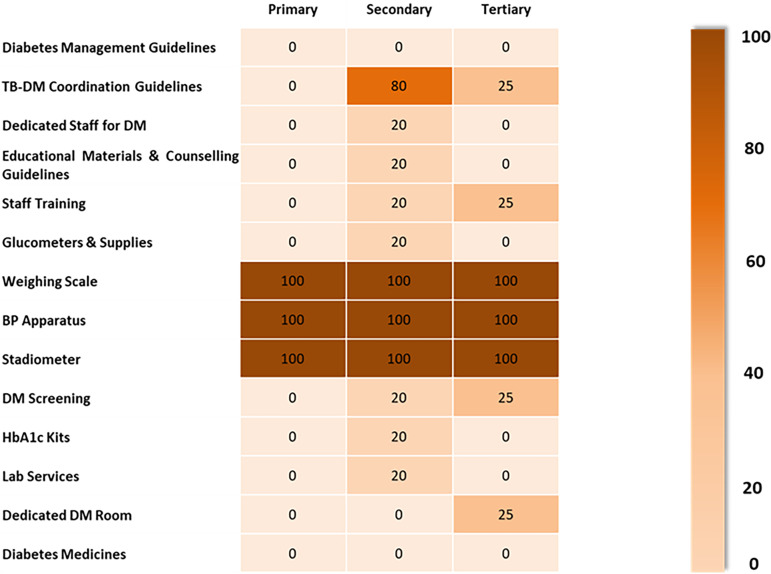
Domain specific readiness for integrated TB-DM care across TB BMUs.

The domain-specific readiness in Figure 5 presents a stark split between general equipment and DM-specific readiness. Basic equipment clustered at 100% across levels, whereas governance and DM-specific tracers (DM-specific guidelines and standard operating procedures (SOPs), training, focal person, glucometer/strips, HbA1c access, DM medicines) were not available, with only isolated pockets at 20–25% (i.e., one facility within a stratum). Although TB BMUs were embedded within primary, secondary, and tertiary public hospitals, our assessment explicitly targeted BMU-level readiness, that is, what TB staff could deliver within the TB unit rather than the hospital’s broader NCD capacity. Consistent with the heat map, readiness hinged on BMU-specific ‘software’ and DM-specific ‘hardware’: written guidelines/SOPs, a named focal person and recent training, point-of-care glucometers with strips and lancets, access to HbA1c via a defined pathway, and availability of DM medicines. General hospital equipment was often present, yet tertiary placement did not translate into higher BMU readiness because these TB units lacked the programmatic integration enablers and DM-specific commodities. Integration capacity depended on what was organized and stocked for TB staff in the BMU, not on the nominal hospital level, underscoring the need to institutionalize TB-DM SOPs, commodities, role ownership, and referral arrangements inside the TB programme workflow.

**Table:** 

**High readiness TB BMU – positive deviance summary**
A TB BMU embedded in a leprosy hospital consistently operationalized TB-DM integration within comparable resources. Hospital leadership instituted a mandated random blood glucose (RBG) screening step for TB patients (Implementation Climate → *relative priority*; *compatibility*), the DOTS facilitator was trained in DM screening and cascaded skills to additional staff under supervision (*access to knowledge & information*; *leadership engagement*), and a laboratory services contract ensured sample transport and timely result return (*networks & communication*). A minimal commodity set (glucometer) supported point-of-care screening (*available resources*). Together, these micro-architectures, policy cue, task shifting human resource roles, simple SOP, external lab pathway, explain the site’s superior readiness and form a transferable model for early scale-up across BMUs.

**Figure 6.**
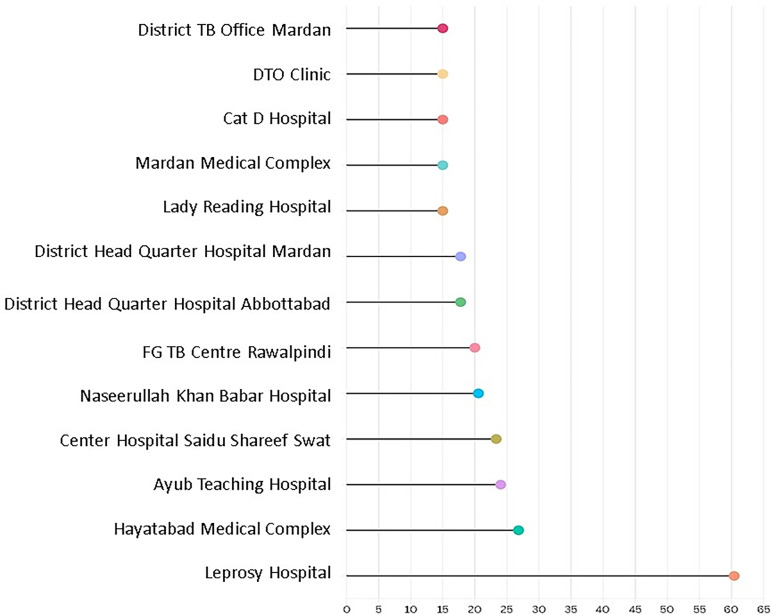
Facility-level composite readiness scores for TB-DM integration.

The lollipop plot displays TB facility-level overall readiness scores, ranked from lowest to highest (Figure 6). However, it does not represent the readiness of the primary, secondary, or tertiary public hospitals within which these TB BMUs are located. This visualization highlights substantial variation across facilities, with readiness scores ranging from 15.0 to 60.4 (21.9 ±12.21).

Following the facility readiness assessment, to move beyond descriptive reporting, we interpreted the qualitative findings through the CFIR inner setting lens using a hybrid inductive–deductive approach. Inductively derived themes from the focus groups were mapped to CFIR constructs to clarify mechanisms underpinning the quantitative assessment gaps and to identify actionable levers for TB-DM integration.

## Access to knowledge & information

This section integrates the themes, guidelines & human resource and health workforce capacity and training gaps. Across sites, limited codification of practice (absence of TB-DM SOPs and guidelines), lack of recent in-service training for integrated care, and lack of a designated focal person for DM screening and diagnosis in TB patients, constrained the capability to adopt and standardize integrated screening and management tasks. These deficits align with low readiness scores in the guidance and human resource domain and indicate that knowledge infrastructure, rather than nominal facility tier, was among the primary barriers to initiating TB-DM integration at the BMU level.

## Guidelines & human resources

The quantitative findings highlighted a near total absence of DM-specific guidelines, dedicated staff, and training across primary TB BMUs (0%) and a minimal presence at secondary and tertiary level BMUs (20–25%).

Qualitative findings reinforced these gaps, with healthcare workers and TB programme managers consistently reporting the absence of formal protocols, structured guidelines, or training mechanisms for integrating DM care into TB services. Overburdened staff, lack of organizational prioritization, and absence of dedicated roles for NCD management were identified as key barriers.

## Health workforce capacity and training gaps

Despite the fact that the TB facilities staff across all districts were aware of the importance of integrating TB-DM care, the existing TB load on the facilities emerged as a significant barrier.

Considering the burden on TB patients across all TB facilities, medical officers and DOTs facilitators are already overburdened. To integrate DM screening, diagnosis, and management, we need both training and additional staff. Currently, counselling services are only available for multidrug-resistant tuberculosis (MDR-TB) patients. Integrating DM care will require training staff for lifestyle modification, nutrition, and mental health counselling and support, along with medical support (Participant 104).

## Available resources

This section integrated basic equipment, inadequate infrastructure and resources, diagnostics, medicines, and funding. Across all the TB BMUs, diabetes-specific commodities (functional glucometers, strips and lancets), HbA1c access pathways, and DM medicines were largely unavailable to the TB staff.

## Basic equipment

Qualitative data showed the universal availability of general equipment (weighing scales, BP apparatus, stadiometers), which also presents the adherence to TB treatment protocols, but significant gaps in DM-specific tools (glucometers, strips), absent in primary and tertiary BMUs, and present in only 20% of secondary BMUs.

Qualitative findings mirrored this as the participants reported that the TB BMUs are adequately equipped for TB management, irrespective of the level of care. The participants reported a lack of DM-specific diagnostic tools, highlighting that structurally and operationally, the basic management units were not equipped to support NCD integration within routine TB care. Beyond availability, participants highlighted functionality and maintenance issues. While general equipment was universally available, DM-specific tools were absent due to procurement limitations within the current TB Control Program budget allocation and lack of integration protocols.

## Inadequate infrastructure and resources

Facilities frequently lack the necessary infrastructure, such as rooms for DM screening and diagnosis. Diagnostic tools like glucometers were not available to TB staff, limiting the feasibility of offering integrated care.

TB is managed under a vertical program, so we are structurally and operationally equipped for TB screening, diagnosis, and management. However, so far there is no integration of NCD in Pakistan in the TB Control Program, so the majority of the TB staff have no screening or diagnostic equipment in this regard (Participant 113).

## Diagnostics

Quantitative findings reported no availability of DM screening or diagnostic services at the primary care level BMU, and minimal availability (20–25%) at secondary and tertiary levels for HbA1c testing, lab services, and screening activities.

Qualitative findings confirmed these results, highlighting the absence of systematic screening protocols, insufficient diagnostic capacity, and reliance on informal, fragmented referral pathways. Participants described these gaps as barriers to continuity of care and timely diagnosis.

Primary care facility near my house is the place I go for TB medicine; however, once or twice I asked the TB staff to check my sugar as I was feeling dizzy, but they had no basic machine to check. If I go to a nearby pharmacy or any private clinic, they charge so much, and I barely have any earnings to pay every time. If a sugar machine (glucometer) is given to staff, it can help poor people like us (Participant 109).

The absence of basic DM diagnostics like glucometers was attributed to supply chain limitations, budget constraints within vertical TB programming, and a lack of policy directive for TB facilities to provide NCD services.

## Medicines

Quantitative findings identified a complete absence of DM medicines availability with TB DOTS and MO across all facility levels (0%).

Qualitative findings further illustrated the consequences of this gap, with patients and providers highlighting financial burdens, fragmented care pathways, and the inefficiencies of seeking DM treatment separately from TB services. Staff highlighted the need for system-level planning for integrated pharmaceutical provision.

## Financial constraints

Financial constraints emerged at both the system and patient levels. The current TB programme fully supports TB screening, diagnosis, and management efficiently and effectively within the limited available financial resources. However, integrating DM care requires additional funding.

For the patient, financial barriers are pervasive. Patients reported financial burdens of varying nature.

Being a hypertensive and diabetic patient, I seek treatment from a local GP near my house, but for TB, I am getting treatment from the TB facility. With limited earnings, paying GP fees and buying medicines for hypertension and diabetes mellitus get me into financial problems. Sometimes I take a loan from family or friends. If hypertension and diabetes mellitus treatment are available at the TB centre, it can save me money (Participant 118).

The inability to afford diagnostics and medication for DM further exacerbates patient vulnerability, complicating the successful management of both conditions.

## Relative priority

Within CFIR, relative priority captures how strongly a new practice is valued compared with competing demands. Since TB is managed under a vertical programme, without clear prioritization cues for comorbidity-integrated care, screening remained sporadic. To raise its relative priority, TB-DM screening in the routine workflow with defined indicators is a must.

## Weak organizational priority for integrated care

Integrated TB-DM care is not yet institutionalized as a strategic priority within national and provincial TB programmes. As a result, integration efforts are piecemeal and driven by individual champions rather than organizational mandates.

In Pakistan, TB is managed under the vertical program, as our colleague has already mentioned. There were a few research projects for TB comorbidity, but once the project ends, the funding also ends. We have not seen any document or heard anyone talking about TB comorbidity integration for actual implementation (Participant 105).

Weak organizational priority was primarily due to broader structural constraints, not due to individual managerial or facility problems. Pakistan’s TB programme is vertical, and its mandates and performance are primarily TB outcomes. The fact that no policy implementation, financing systems, or effective ways exist to handle comorbidities means that diabetes care is left on the periphery of routine TB services. Since the process of integrating TB and diabetes is not institutionalized; it is only done occasionally and is largely financed by research projects that are donor-funded.

## Absence of a formal screening mechanism for comorbidity in TB patients

Participants consistently reported the absence of formalized protocols and systems to screen TB patients for co-existing conditions in majority of the TB BMUs, particularly DM.

There is currently no mechanism in place to screen patients for multimorbidity, and screening practices rely heavily on individual healthcare providers’ discretion without standardized guidelines or monitoring systems in place in TB BMUs (Participant 101).

Qualitative findings also revealed that the lack of screening mechanisms stemmed from three primary factors: (1) absence of policy mandates at the provincial level, (2) vertical programme structure that discourages cross-disease integration, and (3) performance indicators that focus solely on TB outcomes rather than comorbidity management.

## Network and communication

This section draws together a weak referral system and interdepartmental coordination, and a lack of multi-sectoral collaboration. The absence of written referral SOPs for comorbidities, named contact points, transport/logbooks, and feedback routines produced fragmented handoffs to non-TB services (e.g., HbA1c), which is reflected in the heat map’s high barrier intensity for coordination and execution.

## Weak referral system and interdepartmental coordination

The absence of structured referral pathways between TB and DM services emerged as a major impediment. Existing practices rely on informal referrals through verbal communication rather than systematic, protocol-driven processes.

Referral systems can be developed, but at the moment they are informal and inconsistent (Participant 107).

We don’t have lab services and blood sample transportation mechanisms at most of the TB BMUs. For the integrated care, the program will require additional funds to establish lab referrals, sample transportation, riders, and other necessary logistic arrangements (Participant 114).

The lack of screening and diagnosis mechanisms leads to fragmented care and increased patient comorbidity burden due to the need to navigate between uncoordinated services independently.

Participants identified specific barriers to referral system development: (1) absence of formal interdepartmental agreements, (2) lack of dedicated coordination roles, (3) insurance policy gaps that don’t cover integrated services, and (4) absence of financial incentives for cross-referrals.

## Lack of multi-sectoral collaboration

Although participants recognized the importance of multi-sectoral collaboration, there was no evidence of established mechanisms to support this. Leadership engagement across sectors, including healthcare providers, community leaders, the pharmaceutical industry, and civil society, is weak in Pakistan, leading to isolated efforts and duplication of services rather than coordinated, comprehensive strategies.

## Implementation climate

We coded deficiencies in TB-DM comorbidity data under implementation climate, primarily the sub-constructs goals & feedback and learning climate.

## Deficiencies in the comorbidity data collection and reporting system

Participants noted that existing data systems, including Tuberculosis Treatment Facility Card (TB-01), Tuberculosis Patient Card (TB-02), Tuberculosis Care Facility/District TB Register (TB-03), and District Health Information System, are not fully designed to capture the full spectrum of information related to DM comorbidity in TB patients beyond documenting DM primarily as a risk factor. This omission limits the ability to monitor, evaluate, and improve integrated care practices. The lack of integrated data systems creates a significant blind spot for programme management and policymaking.

## Convergence and divergence analysis

Based on the analysis, there was clear and consistent convergence between qualitative and quantitative data; however, there was no notable divergence between data. Formal guidelines, staff training, and dedicated personnel were absent; general equipment was present, but diabetes-specific tools were lacking; screening and diagnostic readiness were minimal to non-existent; and TB services did not stock DM medicines.

## Mapping barriers to the CFIR inner setting domain

We employed the mapping of barriers to the CFIR inner setting domain to strengthen the thematic analysis rigour by providing a validated lens through which to examine the complex interplay of factors influencing implementation outcomes. Moreover, our aim for mapping findings to CFIR was also the identification of actionable targets for future intervention and informing strategies that can be contextually tailored to address the specific inner setting dynamics of the TB health facilities. The barriers identified in our study align closely with constructs within the Inner Setting domain of CFIR, including structural characteristics, networks and communications, culture, implementation climate, available resources, and access to knowledge and information (*CFIR)*.

**Figure 7.**
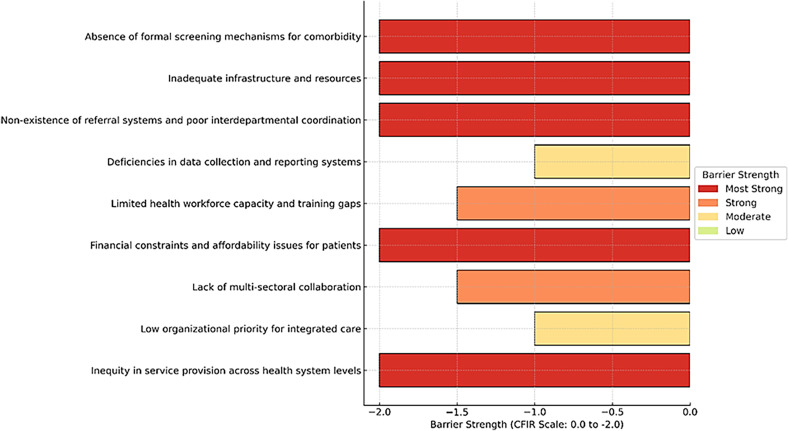
Heat map of integrated Diabetes care implementation barriers at TB BMUs.

We generated a heat map based on the CFIR index (Mehret Assefa, 2018) to enhance the utility and clarity of findings and to provide a visual representation of the strength and severity of barriers especially for the stakeholders, policy makers and implementers (Figure 7).

Heatmap summarizes CFIR-coded barriers to TB-DM integration at the TB BMU level, indexed on a 0 to −2 severity scale (0 = none; −2 = most severe). The most severe barriers clustered within the inner setting and execution. This included the absence of a formalized TB-DM screening step, inadequate infrastructure and supplies, and non-functional referral pathways with poor interdepartmental coordination, patient financial burden, and inequities across levels of care. Strong barriers (−1.5) reflected capability and engagement deficits, including limited workforce capacity, training, and weak multi-sectoral collaboration, while moderate barriers (−1.0) captured underdeveloped enabling systems in routine data collection and reporting, and a low organizational priority for integration.

## Discussion

We employed a theory-informed mixed-methods design that integrated WHO-SARA readiness assessment with CFIR-guided qualitative analysis to deliver a comprehensive, systems-level evaluation of TB BMUs readiness for integrated TB-DM care in Pakistan. Of the 13 TB facilities assessed, only one demonstrated high readiness, while the remaining twelve exhibited low readiness across key service domains for integrated TB-DM care and service delivery.

This mirrors the low readiness patterns identified in previous TB health facility readiness assessments for DM management in other low-and-middle-income countries like Tanzania, where readiness was 10.8% despite 70% availability of DM services (Shayo and Shayo, 2021). Aligning with the Tanzanian experience, our study also highlighted the overburdened existing health workforce as a barrier to integration, consistent with previous studies (Almossawi et al., 2019), along with overall low readiness across all four domains. Similarly, Bangladesh reported only 36.3% of facilities meeting high readiness thresholds, despite nearly universal equipment availability, highlighting critical weaknesses in staffing, diagnostics, and medicines(Rafi et al., 2024). Findings from a study conducted in Ethiopia highlighted the limited availability of integrated care 25% of surveyed health facilities offered integrated TB and DM care, with an overall integration rate among patients of 13.4% (Salato and Setswe, 2024).

The low readiness of primary care TB facilities in our study was in line with the studies conducted in Bangladesh, where the root-level facilities, like the community health centres, lacked facilitation to provide the basic management to the DM patients (Biswas et al., 2018; Chowdhury et al., 2022). Our findings also echo those reported in the wider literature on TB and other communicable–NCD integration, where health facilities frequently exhibit integration and implementation determinants and limitations (Ojo et al., 2019; Shayo and Shayo, 2021; Chamba et al., 2022; Foo et al., 2022; Milice et al., 2024; Kakisingi et al., 2025). While these studies often describe the operationalization of Level 1 or Level 2 integration models, the recurring themes of poor infrastructure, weak referral systems, insufficient diagnostic capacity, and fragmented service delivery are consistent across these diverse LMIC settings. However, an important element of readiness assessment is the availability of basic equipment across all the facilities, which projects the strengthened TB service delivery consistent with other studies (Farzadfar et al., 2012; Kakisingi et al., 2025). There is considerable variability in the levels of integration observed across different settings, with some higher-level urban facilities demonstrating greater capacity and readiness than their rural and primary care counterparts (Rafi et al., 2024). This uneven distribution of readiness highlights the disproportionate impact on vulnerable populations in rural regions, where infrastructural and operational constraints contribute to poorer health outcomes (Nyirenda et al., 2023). However, this trend varied in our study as all primary care BMUs that are mostly situated in or near rural populations exhibited consistently low levels of preparedness to DM related services, which was also reflected in secondary and tertiary institutions, with the exception of one well-performing location. The TB BMUs across all tiers of health facilities ensure a high level of standardization and effective functioning of the TB service. Since the services of DM are not institutionalized and integrated into the TB platform yet, essential DM-specific input guidelines, diagnostics, medicines, trained personnel, and referral mechanisms were always missing. The readiness gaps presented in the BMUs do not reflect areas of weakness in the provision of TB services but rather reflect a lack of integrated structures in the system of TB and DM across each facility, regardless of whether it is rural, peri-urban or urban.

Facilitators of successful integration are multifaceted and include factors such as the adoption of standardized clinical algorithms, effective policy frameworks, and the use of innovative technologies, including mobile health and telemedicine, to bridge resource gaps. Moreover, targeted capacity-building initiatives ranging from focused training programmes for healthcare workers to the development and dissemination of updated treatment guidelines have demonstrated potential in enhancing health facility readiness for integrated TB-DM care (Salifu et al., 2021)

The WHO and International Union Against Tuberculosis and Lung Diseases established a collaborative framework for care and control of TB and DM as both diseases affect the treatment outcomes of each other and generate a substantial burden on the health system, especially in LMIC like Pakistan. The significance of our study findings cannot be overstated. Integrated TB-DM care is increasingly recognized as essential for addressing the dual burden of communicable and NCDs in LMICs, yet our study demonstrates that the capacity to implement these models is overwhelmingly insufficient. The stark reality is that only one out of thirteen facilities in our setting had the foundational readiness required to operationalize such integration effectively. This finding is emblematic of broader systemic challenges documented across LMICs and reinforces the need for targeted investments in human resources, clinical infrastructure, diagnostic systems, supply chain management, and health information systems, as without deliberate investment in system strengthening, true readiness for integrated TB-DM care will remain elusive.

Furthermore, our heat map analysis provides a novel and visually impactful synthesis of these barriers, highlighting the most critical areas requiring immediate attention such as the absence of referral systems, inadequate infrastructure, and limited human resources. These findings align with global evidence that underscores the need for comprehensive health system strengthening before integrated TB-DM care can achieve its intended impact.

## Implication for policy

Addressing the low readiness barriers in Pakistan requires moving beyond broad recommendations for investment to targeted, actionable strategies. National TB and NCD programmes should integrate facility readiness assessments for comorbidities into routine supervision and monitoring, ensuring that service expansion is matched by real capacity gains. Donors and development partners must recognize that achieving integrated care requires investments in health system infrastructure, human resources, and referral networks, not just disease-specific interventions. Readiness metrics aligned with CFIR or WHO-SARA tools should inform resource allocation, focusing efforts where gaps are most severe. Moreover, health information systems should incorporate indicators of service integration and readiness to enable ongoing tracking and course correction.

## Strengths and limitations

The key strength of this study is its rigorous application of an established implementation science framework (CFIR) to analyse barriers and facilitators at the facility level, combined with a visual heat map to facilitate interpretation and priority-setting. This was a mixed-method study focusing on readiness assessment of thirteen public TB BMUs in five districts of Khyber Pakhtunkhwa and Punjab. Facilities were not sampled to be statistically representative of the whole of Pakistan or managing authority (the private sector was not included); accordingly, findings should not be interpreted as national estimates but as hypothesis-generating for early implementation in comparable public BMU settings. We did not measure economic costs or formal implementation outcomes (acceptability, feasibility, fidelity, penetration, sustainability) as this was not within the scope of this study. As such, the study identifies *what* is missing to enable TB-DM integration but cannot quantify *how much* it would cost.

Furthermore, while our analysis identifies inner setting determinants, it does not capture or report the outer setting factors (e.g., policy environment, funding structures), which undoubtedly influence facility readiness and functioning of provincial TB programmes in Pakistan.

## Conclusion

The findings reinforce that the majority of the facilities providing exceptional TB care are not fully prepared to integrate DM care, with clear patterns of deficits in infrastructure, human resources, referral systems, and implementation climate. Our findings, despite being consistent with broader literature, provide first-hand evidence for the implementer and policy makers to map domains requiring strengthening for TB-DM integration.

## Supporting information

10.1017/S146342362610111X.sm001 Aleem et al. supplementary material Aleem et al. supplementary material

## Data Availability

The dataset analysed in this study is available from the corresponding author upon reasonable request.

## Supplementary material

The supplementary material for this article can be found at https://doi.org/10.1017/S146342362610111X.
