# Supplementary material for: From silos to synergy: assessing tuberculosis basic management units readiness for integrated diabetes mellitus care across different districts of Pakistan
Source: Prim Health Care Res Dev. 2026 Apr 10;27:e47. doi: 10.1017/S146342362610111X (PMC13080535; doi:10.1017/S146342362610111X)
Supplement: Aleem et al. supplementary material [file S146342362610111Xsup001.docx]

Annexure A

**Positionality and functioning of TB BMUs across levels of care in Pakistan**

In Pakistan’s National TB Control Program (NTP), the Basic Management Unit (BMU) is the operational node for case detection, treatment initiation and follow-up, recording/reporting (TB-01/02/03), drug logistics, and referral. BMUs are embedded within primary, secondary, or tertiary public facilities but operate under standard NTP routines, linked to the provincial TB program for supervision, medicines, diagnostics networks (microscopy/GeneXpert), and quarterly cohort reporting. While the program processes are standardized, the position within the health system—and thus the catchment, patient mix, and available co-services—varies meaningfully by level and urban/rural context.

**Primary-care BMU (BHU/RHC/stand-alone TB clinic).**

Typically situated in rural and peri-urban communities and serving local catchment (e.g., union councils and adjacent villages), primary BMUs are the first contact for presumptive TB. Core functions include symptom screening in OPD, sputum collection with on-site microscopy where available (or sample transport to an Xpert site), treatment initiation for drug-susceptible TB, DOTS/treatment support (often leveraging Lady Health Workers or family supporters), contact screening, and routine recording/reporting. Co-morbid conditions and complications are referred upward. Staff usually includes a medical officer, DOTS facilitator/treatment supporter, and access to a laboratory technician (shared or visiting); diagnostic breadth is limited, and access to radiology or HbA1c typically requires referral.

**Secondary-care BMU (THQ/DHQ hospitals)**

Located in tehsil or district towns with a broader catchment than primary care, these BMUs manage higher volumes and more complex presentations. They commonly have on-site chest X-ray and, at many DHQs, GeneXpert capacity; laboratory services are more reliable, and pharmacy/logistics are better staffed. Secondary BMUs receive referrals from primary facilities, initiate and monitor treatment, and coordinate onward referral (e.g., to PMDT/tertiary services) when complications, drug resistance, or co-morbidities require it. Clinical oversight may include visiting or posted pulmonologists/MOs; however, BMU delivery still follows NTP registers and supervision, and integration of non-TB services depends on local arrangements.

**Tertiary-care BMU (teaching/referral hospitals)**

Based in urban referral centers with the largest, multi-district catchment, tertiary BMUs manage complicated TB (extra-pulmonary, co-morbid, severe disease) and act as gateways to PMDT sites for drug-resistant TB. They generally have ready access to radiology, specialist input, and GeneXpert (and linkages to reference labs for culture/DST where applicable). Despite this higher resource environment, the BMU remains a program unit: registration, cohort reporting, and drug management follow the same NTP standards as at lower levels. Access to non-TB diagnostics/ therapies (e.g., diabetes services) is often facility-available but not program-owned, so effective use by the BMU depends on SOPs, role clarity, and referral/data pathways rather than nominal hospital tier.

**Urban–rural differentiation and catchment realities**

Primary BMUs predominantly serve rural/peri-urban populations with greater transport and affordability barriers, making reliable sample transport, predictable drug supply, and community-based DOTS critical. Secondary BMUs serve mixed urban–peri-urban catchments with improved diagnostic access but persistent bottlenecks in cross-departmental coordination. Tertiary BMUs serve urban and referred rural patients; while co-services exist, programmatic access requires explicit pathways (forms, named contacts, transport, data fields). Across levels, the functional differentiation is therefore less about the address of the BMU and more about:

1. scope of on-site diagnostics,
2. referral/coordination architecture (networks, SOPs, logs),
3. supply-chain reliability, and
4. data systems that enable monitoring and accountability.

This positionality underpins our analysis and explains why BMU-level readiness can remain low even in tertiary settings.
